# Locke’s National Monthly

**Published:** 1872-11

**Authors:** 


LOCKE'S NATIONAL MONTHLY.—The first number of this new magazine has come to hand, and amply fulfils all expectations as to its character. The articles, original and selected, are all in good taste and show that the publishers have an able corps of contributors. In some of the pieces we recognize the original humor of the editor, Mr. D. R. Locke (Nasby). The low price of this magazine ($1) will place it within the reach of all, and its real merit ought to secure it a large circulation.

